# Compartment syndrome of the leg after thyroid hormone withdrawal; two cases and a systematic review of the literature

**DOI:** 10.1186/s12902-020-00555-y

**Published:** 2020-06-05

**Authors:** Nicole M. van Veelen, Stefan Fischli, Frank J.P. Beeres, Timo Eisenhut, Reto Babst, Christoph Henzen, Björn-Christian Link

**Affiliations:** 1 Department of Orthopedic and Trauma Surgery, Luzerner Kantonsspital Luzern, P.O. Box, Spitalstrasse, 6000 Lucerne, Switzerland; 2 Department of Endocrinology and Diabetes, Luzerner Kantonsspital Luzern, P.O. Box, Spitalstrasse, 6000 Lucerne, Switzerland

**Keywords:** Compartment syndrome, Myxedema, Thyroid gland, Hypothyroidism, Rhabdomyolysis

## Abstract

**Background:**

Acute compartment syndrome is a rare complication of severe hypothyroidism. If the symptoms are not recognized promptly and treatment initiated immediately, there is a high risk of permanent damage. Only few other cases of compartment syndrome due to hypothyroidism have been published and the exact pathophysiological mechanism remains unknown.

**Case presentations:**

A 59 year old male developed acute compartment syndrome of his right lower leg after thyroid hormone withdrawal prior to radioiodine remnant ablation after total thyroidectomy for follicular thyroid cancer. He underwent emergency fasciotomy of all four compartments of the lower leg. The muscle tissue in the anterior and lateral compartment was necrotic and was therefore excised. The second patient was a 62 year old female with Hashimoto’s thyroiditis, who developed acute compartment syndrome of both lower legs after thyroid hormone withdrawal due to non-compliance. Emergency fasciotomy of all four compartments of both legs was performed. The muscle tissue was viable in all compartments.

**Conclusion:**

Although compartment syndrome due to hypothyroidism is uncommon, it is a complication physicians should be aware of. The majority of reported cases are caused by an acute withdrawal of thyroid hormones and not by undetected hypothyroidism. No previous case of compartment syndrome caused by an iatrogenic hormone withdrawal in preparation for radioactive iodine has been published. However, as shown in this report, it may be beneficial to inform patients of this rare complication prior to hormone withdrawal in preparation for remnant ablation after thyroidectomy.

## Background

Acute compartment syndrome can have a variety of causes. It is a well-known complication after vascular surgery or trauma. There have been few reports of cases caused by hypothyroidism [1]. To avoid delay in the initiation of appropriate treatment it is important for emergency physicians to be aware of this rare complication. The pathogenesis of compartment syndrome due to hypothyroidism remains unknown. We present two cases of this unusual cause and review the relevant literature.

## Clinical presentation

### Patient 1

A 59 year old male patient presented to the emergency department with pain in his right lower extremity. He first noticed the pain 2 days prior to presentation. The patient denied any trauma to the leg previous to the pain onset. On the day the pain started, the patient had been discharged from another hospital where he had received radioactive iodine for remnant ablation after thyroidectomy for follicular thyroid cancer with a postoperative TNM-stage of pT3apN0 (0/1). For this reason, the thyroid substitution therapy (triiodothyronine 20 μg twice daily), which was initially started after thyroidectomy 4 weeks earlier, had been paused for 3 weeks. Upon discharge the substitution therapy was initiated again. The patient’s family reported a change of character and unusual behaviour since pausing the thyroid substitution therapy. Review of his medical history showed arterial hypertension, which was treated with an ACE-blocker. Furthermore, the laboratory workup prior to thyroidectomy showed elevated anti-thyroid peroxidase antibodies (5734 U/ml) suggesting Hashimoto’s thyroiditis.

**Fig. 1:**
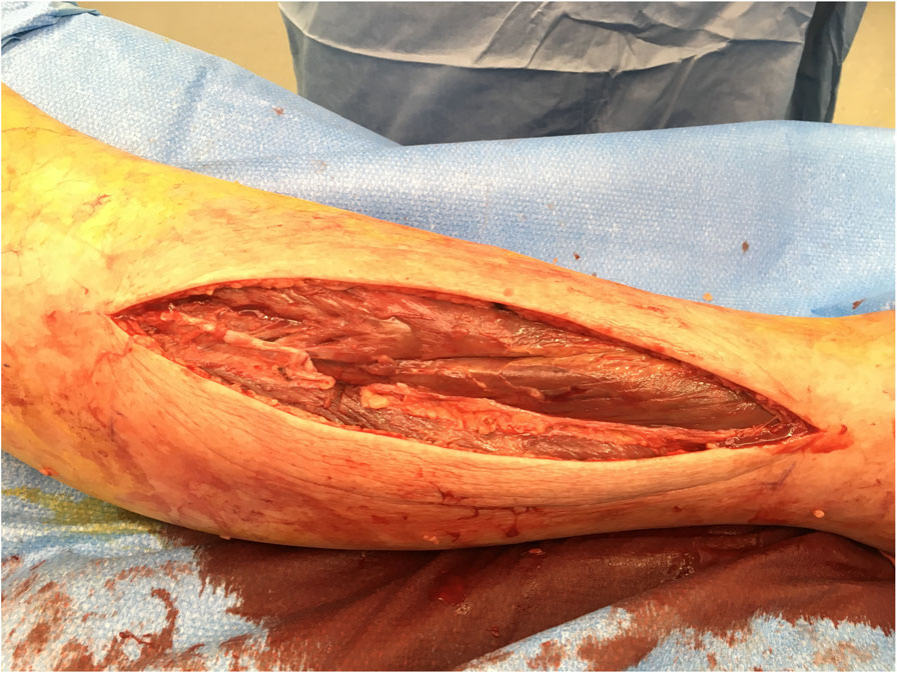
Necrotic tissue in anterior and lateral compartment

Physical examination demonstrated erythema and swelling of the right lower leg. Both Meyer and Hohmann signs where positive, however, Payr sign was negative. Laboratory values were as follows: Leucocytes 15.0 G/l (2.6–7.8), D-Dimer 1555 ng/ml (< 500), Creatinine 109 μmol/L (44–97), lactate dehydrogenase (LDH) 3051 U/L (< 250), c-reactive protein (CRP) 109 mg/L (< 5), creatinine kinase (CK) 71,971 U/L (< 170), thyroid stimulating hormone (TSH) 88.5 mU/L (0.27–4.2), free thyroxine 0.88 pmol/L (12–22), sodium 136 mmol/l (135–148). Besides the change of behaviour and character there were no other signs of myxedema coma such as hypothermia or cardiorespiratory symptoms. The massive elevation of CK was considered to be caused by rhabdomyolysis due to severe hypothyroid myopathy and was treated with intravenous hydration. Deep venous thrombosis was suspected due to the elevated D-Dimer and anticoagulation was initiated. An ultrasound was scheduled for the following day, which then ruled out a deep venous thrombosis. The patient developed a low-grade fever (38.3°Celsius) and laboratory parameters for infection continued to rise (CRP 159 mg/L). Therapeutic anticoagulation was stopped and empiric antibiotic therapy with amoxicillin/clavulanic acid was started, as it was suspected that the symptoms were caused by an erysipelas. The next day the patient showed a loss of dorsiflexion of the foot and reduced sensibility in the area supplied by the superficial and deep peroneal nerve. The measured pressure in the anterior compartment was 95 mmHg and in the peroneal compartment was 120 mmHg. Emergency fasciotomy of all four compartments of the right lower leg was performed. The muscle tissue in the peroneal and anterior compartments was necrotic (Fig. 1), showed no contractility, and was therefore excised. The muscles in the remaining compartments were healthy and viable. There was no sign of infection and the empiric antibiotic therapy was stopped. After surgery the patient was admitted to the intensive care unit for the initial postoperative care. The CK levels quickly decreased, renal function and electrolyte levels remained normal. Peroral levothyroxine treatment was continued leading to a steady fall of the TSH levels. Delayed primary closure of the fasciotomy wounds was achieved after 3 days and hindfoot arthrodesis was performed shortly after to treat the foot drop. The patient was discharged to a rehabilitation center for gait training. He is currently ambulatory with the aid of crutches.

### Patient 2

A 62 year old female was referred to our emergency department by her family doctor. Over the previous days she had been feeling weak with progressive difficulty walking. Additionally, she had noticed swelling of her tongue and face as well as unusual obstipation and a “croaky” voice.

A review of her medical history was significant for diabetes mellitus type 2 (treated with metformin), Hashimoto hypothyroidism substituted with levothyroxine (125μg/day), thalassemia minor, hypertension (treated with an ACE-blocker) and chronic obstructive pulmonary disease (treated with Salmeterol) with a continued nicotine consumption (60 pack years). Over a month prior to her presentation, she had ceased taking all her medication, as her prescription had run out.

Physical examination showed a pulse of 61 beats per minute, blood pressure of 138/77 mmHg and oxygen saturation of 80% on room air, 94% with 2 l O2/minute. There were no pathologic cardiac findings. Her expiration was slightly prolonged and her face was puffy. Laboratory examinations revealed a potassium of 5.6 mmol/L (3.4–4.5), sodium 134 mmol/l (135–148) serum glucose 10.4 mmol/L (3.9–5.8), HbA1c 8.6% (4.8–5.9), CK 4144 U/L (< 170), TSH > 100 mU/L (0.27–4.2) and a free thyroxine of 0.5 pmol/L (12–22). The patient was admitted to the medical ward and levothyroxine substitution combined with hydrocortisone was started. Two days later she presented with progressively decreasing vigilance and global respiratory insufficiency due to chronic obstructive pulmonary disease, myxedema with alveolar hypoventilation and heart failure. The chest x-ray showed left-sided pleural effusion. The patient was transferred to the intensive care unit for further therapy including non-invasive ventilation, inotropic therapy with dobutamine and intravenous substitution of thyroxine and triiodothyronine (100 μg/day and 20 μg/day respectively). The following day the patient complained of pain in both of her calves. On clinical examination the soft tissue of the right lower limb was tense and pain was exacerbated by passive stretching of the muscle. Venous Doppler ultrasound was negative for a thrombotic process. Compartment pressure was elevated both in the right anterior (70 mmHg) and in the peroneal (60 mmHg) compartments with a median arterial pressure of 80 mmHg. Because the intraoperative pressure measurement also showed elevated pressures in the left lower leg compartments (30-40 mmHg) fasciotomy was performed in all 4 muscle compartments of both lower legs. The muscle tissue in the anterior compartment on the right side was slightly pale. All other compartments showed viable muscles with good contractility. Delayed primary closure of the wounds was achieved within 4 days with full muscle recovery. The patient was discharged to a psychiatric hospital 2 weeks after fasciotomy due to a psychosis, which was most likely triggered by the myxedema.

## Discussion and conclusions

Acute compartment syndrome is a well-known problem after trauma or vascular surgery. Other less frequent causes include snake bites, nephrotic syndrome [1], extravasation of contrast medium [2], and as demonstrated in this and few previous case reports severe hypothyroidism [3]. Compartment syndrome is defined as an increased pressure within a closed compartment that causes a disruption of the perfusion of tissue within. If the pressure in the compartment is not released, permanent damage to the soft tissue within can occur, which in turn can cause loss of function and potentially even the loss of the affected limb [1, 2]. The increase in pressure can be due to conditions which either increase the content or decrease the size of the compartment [2, 4].

**Fig. 2:**
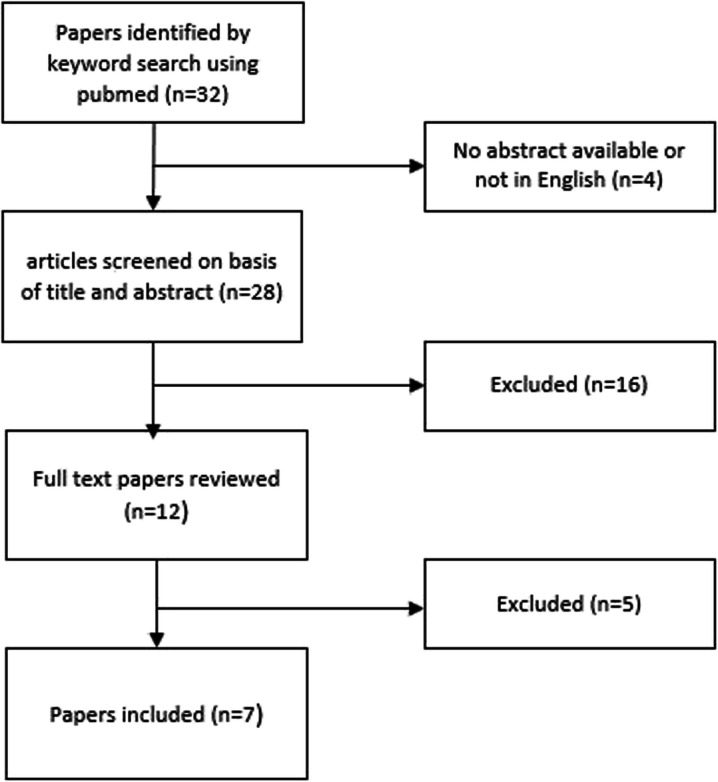
Flow diagram of study selection

To find other cases of compartment syndrome associated with hypothyroidism we performed a Pubmed-search using the combined keywords “hypothyroid*” and “compartment syndrome” working with PRISMA guidelines [5] (Fig. 2). Only publications in English with an available abstract reporting on compartment syndrome with no other cause besides hypothyroidism were included. Reports involving children under 18 years of age where excluded.

We found five other reported cases of compartment syndrome due to hypothyroidism, with the first one published in 1993. (Table 1) An additional case was reported in a patient that had concomitant adrenal insufficiency and one case after coronary artery bypass grafting in a patient with undiagnosed hypothyroidism. We also found one literature review on hypothyroid myopathy [3], but no specific data to compartment syndrome. None of the previously published cases were caused by iatrogenic hormone withdrawal in preparation for radioiodine remnant ablation after thyroidectomy, making our case the first.

**Table 1:** Review of literature

	Year	Age, gender	Cause of hypothyroidism	Laboratory values	Compartment pressure (mmHg) and viability	Unilateral vs bilateral
Thacker A. [6]	1993	40 male	Not mentioned, antibodies normal	CK?	Pressure not mentioned	bilateral
				TSH 118 mU/l	Anterior Compartment released	
Hsu S. [7]	1995	33 female	Undetected Hashimoto	CK 11′319 U/l	Lateral: 65 (necrotic)	unilateral
				TSH 87 mU/mL	Anterior: 124	
					Superf. dorsal: 24	
					Deep dorsal: 62	
Mills J [8]	2010	72 male	Undetected hypothyroidism, aetiology not mentioned	CK unknown	Anterior: 75 mmHg (necrotic)	bilateral
				TSH 42.3 mU/l		
Muir P. [9]	2012	22 male	Undetected Hashimoto	CK > 25′000 U/l	Pressure not messured (MRI shows necrosis of M. tib. Ant.)	bilateral
			+ adrenal insufficiency	TSH unknown		
Hariri N. [10]	2014	60 male	Medication non-compliance	CK 68′000 U/l	Anterior: 75 (necrotic)	Bilateral
			Aetiology of Hypothyroidism not mentioned	TSH 176 mU/L	Lat. &dorsal: 10–15	
Modi A. [11]	2016	42 female	Hashimoto	CK 1854 U/L	Lateral: 142	unilateral
			Medication non-compliance	TSH 147 mU/L	Anterior: 96	
Musielak M. [12]	2016	49 female	Medication non-compliance	CK 13977 U/l	Pressure not mentioned	Bilateral upper and lower extremities
			Aetiology of Hypothyroidism not mentioned	TSH 164 mU/L	Anterior and lateral necrotic	

The exact pathomechanism of compartment syndrome in hypothyroidism is still unknown. Rhabdomyolysis caused by hypothyroidism is a rare condition and is usually triggered or exacerbated by renal or adrenal failure, by excessive exercise or by the consumption of lipid-lowering drugs or alcohol. The pathogenesis remains discussed with possible causes being a reversible defect in glycogenolysis and low mitochondrial enzyme activity leading to mitochondrial metabolism impairment [3]. The edema caused by muscle necrosis could lead to an increase in compartment content resulting in compartment syndrome [13]. However rhabdomyolysis is also a common complication of compartment syndrome [14]. It is therefore impossible to determine whether the compartment syndrome in patients with hypothyroidism is caused by rhabdomyolysis or whether, analogue to compartment syndrome after trauma [14], rhabdomyolysis is a complication of compartment syndrome. As neither of our patients had any risk factors for rhabdomyolysis, such as consumption of lipid-lowering drugs, it seems more likely that rhabdomyolysis was a result of the compartment syndrome.

Other forms of myopathy caused by hypothyroidism include Hoffmann’s syndrome and Koche-Debre’-semelaigne syndrome. These syndromes describes the presence of hypothyroidism with muscle weakness and pseudohypertrophy in adults [15] and children [3] respectively. The Calf muscles (gastrocnemius) are almost always involved [3]. The etiology of the muscle enlargement remains controversial [16]. It has been suggested that it is due to an increase in connective tissue as well as size and number of muscle fibers [15]. Other papers have described glycosaminoglycan deposition and increased extravasation of plasma protein in the interstitial space coupled with impaired compensatory lymph flow and protein return rate [7]. These findings suggest an increase in the volume of the compartment contents [7] which could lead to compartment syndrome. However, as mentioned above, Hoffmann’s syndrome usually involves the gastrocnemius, whereas in our cases and all but one of the other case reports [6, 8–12] we found, the anterior and lateral muscles were predominantly involved. A connection between Hoffman’s syndrome and compartment syndrome therefore seems unlikely. We found no apparent reason for the anterior and lateral compartments to be affected more than other compartments. A larger collection of patients would be needed to be able to further investigate this observation.

Three of the previously published cases [7, 9, 11] and both cases described in this report had Hashimoto’s thyroiditis. A link between hypothyroidism due to autoimmune thyroiditis and the occurrence of compartment syndrome therefore seems possible. However, we found no literature elaborating this possible connection. Furthermore, the antibodies were normal in the first case published [6], ruling out autoimmune thyroiditis in this patient. Although to our knowledge it has not yet been discussed, the majority of cases (three of the previously published cases plus our two cases) of compartment syndrome due to hypothyroidism are caused by a withdrawal of thyroid hormones and not by undetected and untreated hypothyroidism. This raises the question whether patients undergoing hormone withdrawal are at a higher risk of compartment syndrome than those with undetected hypothyroidism. However, the amount of available literature is too limited to draw reliable conclusions.

In summary, although compartment syndrome due to hypothyroidism is uncommon, it is a complication both endocrinologists and emergency physicians should be aware. For if it is missed, the consequences for the patient can be devastating.

The exact pathophysiological mechanism causing this rare condition still needs to be elucidated. Withdrawal of thyroid hormones might play a role in triggering the development of compartment syndrome, but evidence is sparse. Further, autoimmune thyroiditis could influence the development of compartment syndrome, however literature supporting this presumption is lacking. In patients with known hypothyroidism presenting with leg pain, compartment syndrome should be included in the differential diagnosis and the appropriate investigations performed. Moreover, it may be beneficial to inform patients of this rare complication prior to hormone withdrawal in preparation for remnant ablation after thyroidectomy.

## Data Availability

All data generated or analysed during this study are included in this published article.

## Abbreviations

ACE: Angiotensin converting hormone
LDH: Lactate dehydrogenase
CRP: C-reactive protein
CK: Creatinine kinase
TSH: Thyroid stimulating hormone
HbA1c: Hemoglobin A1c
